# Non-selective Separation of Bacterial Cells with Magnetic Nanoparticles Facilitated by Varying Surface Charge

**DOI:** 10.3389/fmicb.2016.01891

**Published:** 2016-12-01

**Authors:** Xin-Lei Gao, Ming-Fei Shao, Yi-Sheng Xu, Yi Luo, Kai Zhang, Feng Ouyang, Ji Li

**Affiliations:** ^1^Shenzhen Graduate School, State Key Laboratory of Urban Water Resource and Environment, Harbin Institute of TechnologyShenzhen, China; ^2^Shenzhen Key Laboratory of Water Resource Utilization and Environmental Pollution ControlShenzhen, China; ^3^State-Key Laboratory of Chemical Engineering, East China University of Science and TechnologyShanghai, China; ^4^College of Environmental Science and Engineering, Ministry of Education Key Laboratory of Pollution Processes and Environmental Criteria, Nankai UniversityTianjin, China

**Keywords:** magnetic nanoparticle, surface charge, microbial community, adsorption, glass fiber filter, bioaerosols

## Abstract

Recovering microorganisms from environmental samples is a crucial primary step for understanding microbial communities using molecular ecological approaches. It is often challenging to harvest microorganisms both efficiently and unselectively, guaranteeing a similar microbial composition between original and separated biomasses. A magnetic nanoparticles (MNPs) based method was developed to effectively separate microbial biomass from glass fiber pulp entrapped bacteria. Buffering pH and nanoparticle silica encapsulation significantly affected both biomass recovery and microbial selectivity. Under optimized conditions (using citric acid coated Fe_3_O_4_, buffering pH = 2.2), the method was applied in the pretreatment of total suspended particle sampler collected bioaerosols, the effective volume for DNA extraction was increased 10-folds, and the overall method detection limit of microbial contaminants in bioaerosols significantly decreased. A consistent recovery of the majority of airborne bacterial populations was demonstrated by in-depth comparison of microbial composition using 16S rRNA gene high-throughput sequencing. Surface charge was shown as the deciding factor for the interaction between MNPs and microorganisms, which helps developing materials with high microbial selectivity. To our knowledge, this study is the first report using MNPs to separate diverse microbial community unselectively from a complex environmental matrix. The technique is convenient and sensitive, as well as feasible to apply in monitoring of microbial transport and other related fields.

## Introduction

The detection and quantification of pathogenic and other emerging microbial contaminants in atmospheric environments have drawn much attention (McEachran et al., 2015; Klein et al., 2016). Due to the low atmospheric microbial density, air sampling still faces major challenges. Currently, common air sampling methods for the harvest of microorganisms include total suspended particle (TSP) samplers (Jeon et al., 2011; Yan et al., 2016), membrane filter samplers (Frankel et al., 2012), and stage impactors (e.g., Anderson N6; Hospodsky et al., 2015). Among them, the TSP samplers equipped with glass fiber filter are most widely used, due to their large sampling rate (∼1.0 m^3^/min; Cao et al., 2014; Jiang et al., 2015).

**GRAPHICAL ABSTRACT:**
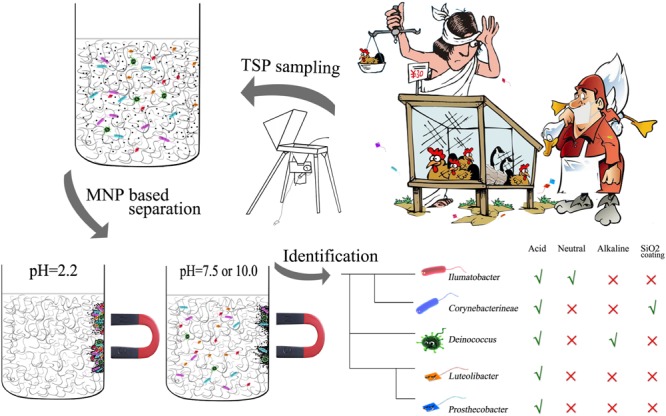
**Schematic representation of the non-selective separation of bacterial cells using magnetic nanoparticles**.

However, broken glass fibers may absorb water several times their dry mass in a wet state because they can swell. Broken glass fibers cause significant difficulties in subsequent DNA extraction, especially when using sonication as the pretreatment step for the detachment of sampled biomass from TSP filters. Small pieces of expanded broken glass fiber filters (less than 50 cm^2^, 10% of the total filter size) clog the extraction tube and hinder subsequent processing steps. Therefore, it has been conventional to use less than 10% of the filter for DNA extraction (Radosevich et al., 2002) in previous airborne microbial studies using TSP samplers; however, they result in a significant loss of sampling throughput and a reduction of detection sensitivity (Yamamoto et al., 2012). Alternatively, sonication based biomass detachment was replaced by a gentle rinse of the filter to avoid breaking glass fibers. By this method, larger filter pieces could be used in DNA extraction, but the DNA recovery was still extremely low due to the low efficiency of initial biomass recovery (Edmonds, 2009).

Magnetic nanoparticles (MNPs) have been applied in the accumulation and removal of microorganisms (Huang et al., 2010), while most of these studies focused on functioning the MNP with functional groups (e.g., vancomycin or mannose) thus to target on specific types of bacteria (Lin et al., 2005; El-Boubbou et al., 2007).

Herein, a convenient MNP assisted method was developed and optimized to efficiently separate microbial biomass from expanded glass fiber pulp. The efficiency and representativeness of the separation method were systematically validated by fluorescent DNA quantification and high-throughput sequencing based community fingerprinting. Unlike the well reported MNP assisted DNA recovery methods (Tanaka et al., 2009; Maeda et al., 2016), the enrichment of microbial biomass achieved in our current study greatly facilitate various whole cell based microbial diagnosis techniques [e.g., fluorescent cell counting (Berney et al., 2008; Ivanova and Dedysh, 2012) and laser capture microdissection (Klitgaard et al., 2005; Tarun et al., 2008)]. This is the first feasible method separating and enriching trace amount of microbial biomass from a complex matrix as far as we known, it is significant for microbial contaminants monitoring, quantification, and other applications.

## Experimental Section

### Air Sampling

Air samples were collected in the Pingshan market in Shenzhen, South China. Samplings were conducted by a high-volume TSP sampler (LS2031, IAT-Laoshan, Qingdao, China). Air was drawn at an average rate of 1.05 m^3^/min for 4 h (polluted air) and 23 h (clean air) resulting in approximately 252 and 1449 m^3^ of flow-through volume. Particulate matters were collected on 20.32 cm^2^ × 25.4 cm^2^ glass fiber filters (PALL, Port Washington, NY, USA) with 99.9% typical aerosol retention. The detail sampling information has been described in Supplementary Table S1. All filters were sterilized by heating at 500°C for 5 h before sampling.

### MNP Assisted Biomass Separation

The citric acid coated Fe_3_O_4_ nanoparticles were prepared by the co-precipitation method as previously described (Lin et al., 2014). The silica encapsulated Fe_3_O_4_ MNPs (Fe_3_O_4_@SiO_2_) were prepared from the hydrolysis and condensation of tetraethylorthosilicate (TEOS; Xu et al., 2006). Subsequently, MNPs were characterized by transmission electron microscopy (TEM) and Fourier transform infrared (FTIR), as shown in Supplementary Figures S1 and S2.

**FIGURE 1:**
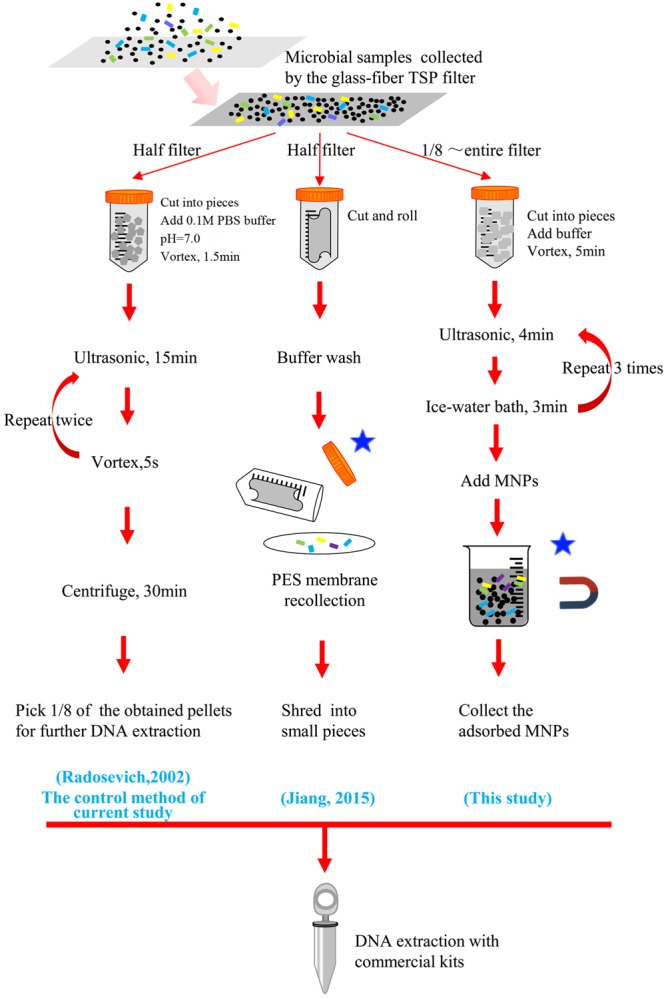
**Illustration of the novel magnetic nanoparticle (MNP) based microbial biomass separation method in comparison with other conventional pretreatment workflow of total suspended particle (TSP) filters**. ^∗^ Steps that may lead to a biased selection of bacterial cells.

To optimize separation efficiency during MNP assisted biomass separation, different buffering pH values (2.2, 7.5, and 10.0) or the introduction of silica encapsulates were evaluated. Briefly, after air sampling a 1/8 section of a TSP filter was placed in a clean beaker containing 40 ml buffer solution. After 30 s sonication for the biomass detachment, the glass filter changed into a pulp slurry. Then, 25 mg of MNPs was added and dispersed with another 30 s sonication. The beaker was gently shaken several times, to facilitate the attachment of MNPs onto microbial cells. Finally, the biomass was recovered through magnetic separation and was ready for the subsequent DNA extraction. In all experiments, the well-recognized conventional method described by Radosevich et al. (2002) was selected as the control method of our current study (**Figure 1**). The relatively small filter size selected here was aimed at a fair comparison with the conventional method control, in which 1/8 section of TSP filters was treated directly with DNA extraction kit without the separation of bacterial cells from glass fiber slurry. Total DNA extraction using the FastDNA Spin Kit for soil (MP, San Diego, CA, USA) was performed following the manufacturer’s instructions. DNA concentrations were determined by fluorescence spectrometer (Hitachi, Tokyo, Japan) using Hoechst 33258 staining dye. The zeta potential of MNPs and bacteria in different pH buffering were measured using Malvern Zetasizer (Malvern instruments Ltd., UK), Specifically, the pH buffer solutions were 0.2 M of Na_2_HPO_4_ (pH 7.5), 0.2 M NaH_2_PO_4_ supplemented with 0.1 M citric acid (pH 2.2), and 0.1 M of Na_2_CO_3_ fixed with 0.1 M NaHCO_3_ (pH 10.0).

### PCR Amplification, Illumina MiSeq Sequencing, and Sequence Analysis

Bacterial DNA was amplified with primer pairs targeting the V4 region of the 16S rRNA gene (F515/R806; Albertsen et al., 2012). Barcodes and linker sequences that allow sample multiplexing during MiSeq sequencing were incorporated at the 5′ end of the forward primer (Zhou et al., 2011). The PCR amplification was conducted in an i-Cycler (Bio-Rad, Richmond, CA, USA). The PCR products from different samples were quantified using a Qubit 3.0 (Thermo Fisher Scientific, Waltham, MA, USA), mixed accordingly to achieve the equal concentration in the final mixture, and then sent to Macrogen Inc (Seoul, Korea) for the high-throughput sequencing on Illumina MiSeq platform. The next generation sequencing data were deposited in SRA database with the submission number of SUB1628949.

In total, 310,000 bacterial sequence reads (∼15,000 reads per sample) were recovered and subjected to MOTHUR software for all subsequent analyses (Schloss et al., 2009). The sequences were trimmed for the removal of primers, barcodes and sequences with ambiguous nucleotides, long homo-polymers or sequence length shorter than 200 bases. For the phylotype analysis, sequences were aligned, examined for chimeras, filtered, and finally classified taxonomically using the SILVA bacterial reference (Pruesse et al., 2007). For the OTU based analysis, aligned sequences were treated with the “dist.seqs” function to generate a distance matrix. Then, the “cluster” command was deployed to construct operational taxonomic units (OTUs) with cutoffs of 3 and 6% dissimilarity.

The target antibiotic resistance genes (ARGs) were quantified using QuantStudio 6 (ABI, Vernon, CA, US), the experimental conditions consisted of an initial denaturation for 5 min at 95°C, followed by 35 cycles of denaturation at 95°C for 15 s, annealing at 60°C for 30 s, and extension at 72°C for 30 s. The annealing temperature and primers are listed in Supplementary Table S2.

## Results

### Design of the Bacterial Separation Method

Development of a convenient method to separate bacterial cells from glass fiber pulp for the full capacity of TSP sampling is an urgent problem. Two major criteria for any potential separation methods are: (1) high efficiency, to eliminate the biomass loss; and (2) avoiding bias, to guarantee a similar microbial composition between original and separated biomass.

To realize the above criteria, citric acid coated Fe_3_O_4_ MNPs were utilized as the competing particle to scavenge biomass from glass fiber pulp. The design of our method in comparison with previous pretreatment methods is illustrated in **Figure 1**. It is believed that citric acid coated Fe_3_O_4_ MNPs have high surface area (Deng et al., 2008) and should be suitable for biomass adsorption. Different buffering pH and particle types were tested to optimize the biomass recovery, including bare Fe_3_O_4_ MNPs in neutral, acidic, and alkaline buffering solutions as well as the silica encapsulation of the MNPs. For a fair comparison, a 1/8 section of a filter membrane (∼60 cm^2^) was used in all extraction experiment corresponding to the maximum capacity of control method shown in **Figure 1** (Radosevich et al., 2002). It should be noted that unlike the two newly developed methods shown in **Figure 1**, there were no separation of bacterial cells and filter fiber slurry was included during the whole workflow of the control method, thus no bias in the selection of bacteria could be guaranteed under this condition.

### The Separation Efficiencies under Various Conditions

**FIGURE 2:**
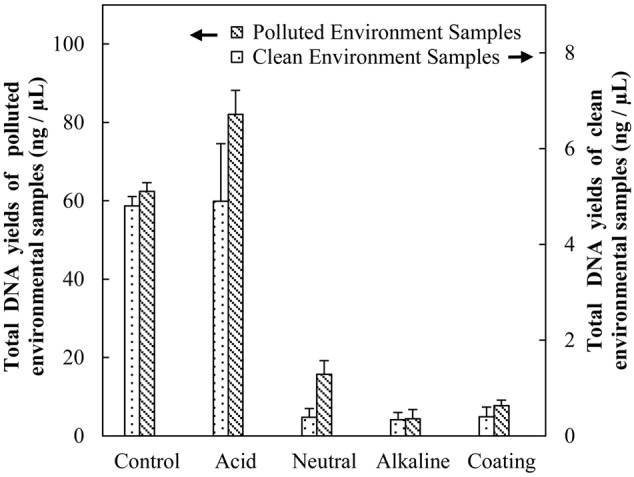
**Evaluation the efficiencies of biomass separation by DNA yield**.

The ultimate DNA yields were used to quantify the separation efficiencies, as shown in **Figure 2**. Among the four treatments tested, Fe_3_O_4_ MNPs in acidic buffer resulted in the highest bacterial recovery. Under acid buffering conditions, the DNA recoveries of MNP methods were comparable to the conventional method control for both clean and heavily polluted air samples. Thus, much more DNA could be easily extracted by simply applying larger piece of the glass fiber filter during MNP assisted biomass separation.

### Selectivity of MNPs against Different Microbial Species

To evaluate the possibility of any biased selection during MNP assisted biomass separation, the extracted DNA of different methods were compared with control method by their microbial community structures. Then, based on the OTUs distribution information, a co-linearity analysis was performed to quantify the similarity of microbial community structure between the different treatments, following Xia’s method (Xia et al., 2010). Briefly, the vector representing each of the five treatments was embedded in the N-dimensional space. N represents the total OTUs numbers present in all five samples. The angle between the vectors represents a measure of relatedness of different communities. The angle between acid buffered Fe_3_O_4_ MNP treatment and the conventional method control group was the smallest one [16.97° (cutoff = 0.03), 16.54° (cutoff = 0.06)] of the four treatments tested in the current study, as shown in **Table 1** and also in Supplementary Figure S4. In addition to the heavily contaminated air sample near live poultry, the method was also used on a relatively clean ambient air samples outside the wet market. Similar small angels (**Table 2**) resulted between the acid buffered Fe_3_O_4_ MNP treatments and the controls, indicating good reproducibility of the present method. These angle values were also compared with some typical sample pairs, including the same DNA with different PCR amplifications (22.3°) or the same biomass with different DNA extractions (14.8°), as shown in **Table 2**. The quite small angel between Fe_3_O_4_ MNPs in acidic buffer treatment and control group indicated a high similarity of microbial communities and thus validated the unselective nature of the present method.

**Table 1:** Co-linearity analysis showing the microbial composition similarities between magnetic nanoparticle (MNP) separated biomass and control methods.

Samples (Treatments)	Angles between two typical treatment (degrees)								
	Cutoff = 0.03 level					Cutoff = 0.06 level				
	1	2	3	4	5	1	2	3	4	5
1	0	16.9	67.2	70.5	78.3	0	16.5	59.5	68.2	77.2
2		0	62.8	72.2	79.0		0	57.7	70.2	78.2
3			0	75.8	79.4			0	72.1	79.3
4				0	20.7				0	17.8
5					0					0

*Treatments: 1, Control; 2, Acid; 3, Neutral; 4, Alkaline; 5, SiO_*2*_-Coating.*

**Table 2:** Co-linearity analysis of typical sample pairs showing their microbial composition similarities.

Sample pairs	Angles between sample pairs (degrees)	
	Cutoff = 0.03	Cutoff = 0.06
Acid MNPs separation and control method (ambient air sample 100 m outside)	24.5	18.8
Acid MNPs separation and control method (ambient air sample 400 m outside)	19.4	16.5
The same DNA with different PCR amplifications	22.3	16.7
The same sample with different DNA extraction and PCR amplification	14.8	14.3
The same media, different sampling points (air sampled at 100 and 400 m outside of the wet market)	65.8	60.7
Different media (ground dust and coastal water)	70.7	68.3
Different media (ground dust and indoor air)	78.5	76.6
Different media (coastal water and air)	82.9	81.6

## Discussions

### Surface Charge as the Main Factor Determining Microbial Selectivity

The bioaerosol samples collected in the current study harbor a diverse microbial community, which could be reflected by the rarefaction curve shown in Supplementary Figure S3. Facing such a complicated mixture of microorganisms, the MNP based methods in the acid buffer condition were successful and unselective in nature, which was comparable with duplicated PCR amplification of the same DNA sample.

**FIGURE 3:**
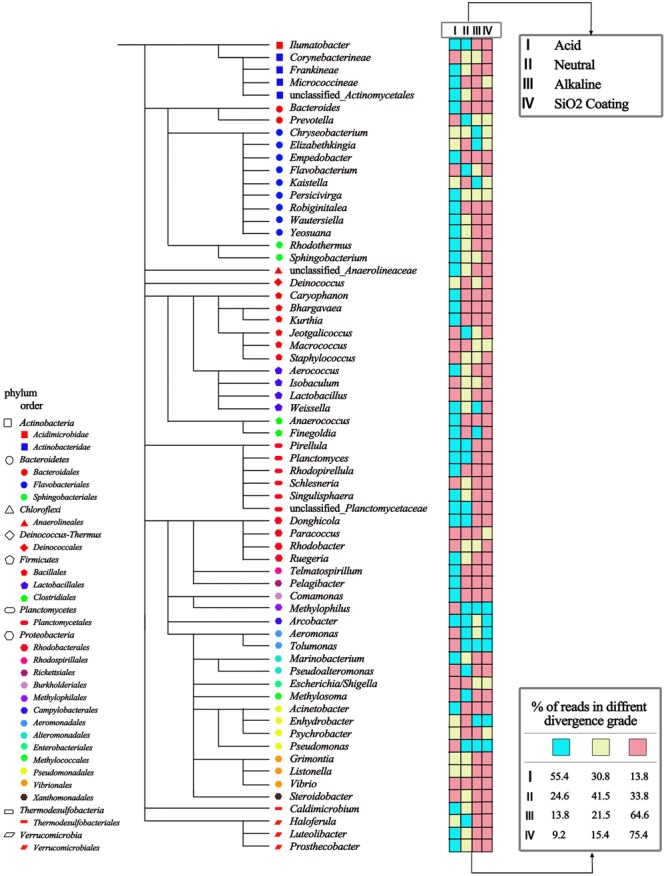
**A phylogenetic tree showing the exact deviations in relative abundance between four MNPs based treatments and control method at class levels**. The divergences in relative abundance were classified into three grades, and indicated by the color chart (cyan: <25% good; yellow: 25∼75% acceptable; red: >75% bad).

For a deeper understanding of the variance of the separated microbial communities by different pH or particle coatings, a phylogenetic tree showing the exact deviations from the control method at class levels is shown in **Figure 3**. Among all four treatments, the acid buffered Fe_3_O_4_ MNPs treatment showed the least bias, in which 86% of separated populations showed acceptable recovery compared to the control method.

Buffering pH was a crucial factor for both efficiency and selectivity of biomass separation. At very low pH (pH 2.2), all types of bacteria cells almost completely adsorbed onto the Fe_3_O_4_ MNPs without any discrimination. As the pH was increased to 7.5, fewer bacteria (10∼20% of low pH) were found adherent to the MNPs and extracted by the magnetic force. The same results occurred for pH 10.0.

The two-step theory was well accepted for the understanding bacterial attachment on solid surface (Meinders et al., 1995). The first step involves the bacteria being transported close enough to allow initial attachment, with the forces including van der Waals forces, electrostatic forces, and hydrophobic interactions (Gilbert et al., 1991; Carpentier and Cerf, 1993). The next crucial step is the irreversible attachment process, by the production of exo-polysaccharides or specific ligands, which may complex with the surface (Dunne, 2002). In the transition from reversible to irreversible attachment, various short range forces are involved, including covalent and hydrogen bonding as well as hydrophobic interactions (Kumar and Anand, 1998). Under current experimental conditions, considering the very short Bio-Nano contact time, the reversible initial attachment no doubt dominate the efficiency and selectively of bacterial separation. Thus hydrogen bond, van der Waals and electrostatic forces were considered as the primary interactions between bacterial cells and MNPs according to DLVO theory (Hermansson, 1999).

**FIGURE 4:**
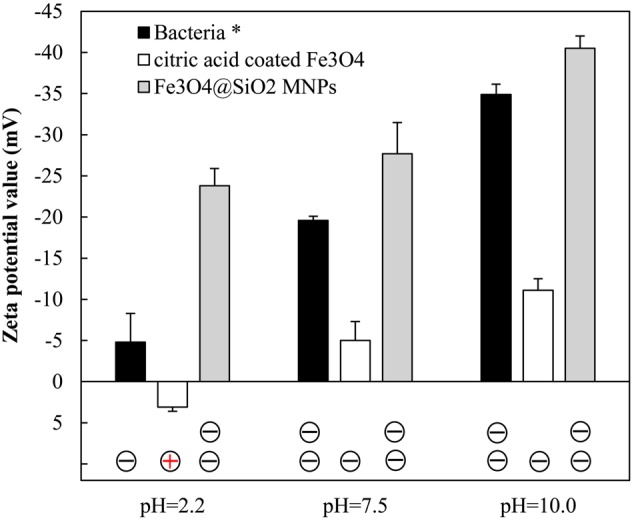
**The Zeta-potential of MNPs and bacteria in different buffering conditions.** ^∗^ For Bacteria samples, multi-peaks could be detected. Only the Zeta-potential values for the dominant peak were plotted.

Apparently, strong hydrogen bonding forms between protonated acid ligands from MNPs and carboxylic or amine groups from the bacteria accounting for the efficient adsorption of all bacteria. However, at higher pH conditions, the electrostatic repulsion between negatively charged carboxylate groups from MNPs and bacteria prevents sufficient biomass adsorption. This mechanism was well supported by surface charge characterization, as shown in **Figure 4**. Under acid buffering, the citric acid coated Fe_3_O_4_ MNP has a zeta potential of 3.1, which is close to zero. As we know the zeta potential measurement by the zetasizer from Malvern is usually a low-resolution method with a standard deviation of ± 5 mV. Therefore, the charge of the electrolyte may convert the zeta potential measurement to negative value; however, the overall result should be very close to zero as determined by the protonation of the carboxylate groups from citric acid at pH 2.2. This is why the hydrogen bonding and van der Waals interactions are critical under such conditions. Interestingly, the microbial cells enriched by Fe_3_O_4_@SiO_2_ MNPs at neutral pH had nearly identical microbial composition with Fe_3_O_4_ MNPs at alkaline conditions (**Table 1**). In both case, significant negative surface charge was demonstrated by Zeta-potential values (**Figure 4**). Considering the low recovery efficiencies (**Figure 2**) of both tests, it is highly likely that only the rare positively charged microorganisms were captured, which again proved that surface charge was the deciding factor for microbial selectivity.

### Significant Implications for the Monitoring of Microbial Contaminants

This is the first report that microbial biomass of low concentration in complex matrix could be easily be separated and enriched for downstream analysis, which has significant implications for trace microbial contaminants detection (e.g., airborne pathogen). Currently, qPCR is still the simplest and most widely used method for the quantification of target genes of interest. According to its definition, the method detection limit (MDL) for the qPCR detection of bioaerosols, is determined by the instrumental detection limit (IDL) of qPCR, the DNA extraction efficiency, and the sampling volume (Hospodsky et al., 2010; Tseng et al., 2015). Among these three factors, the IDL (typically 0.5∼1 copy/μL for a SYBR Green dye based detection) and extraction efficiency (approximately 15.5∼43.3% for most commercial kits; Mumy and Findlay, 2004) are the most difficult to improve further. Thus, most studies focus on novel sampling methods with a higher flow rate to lower the MDL of quantification in bioaerosols.

A high-volume TSP sampler is a desirable method because its sampling rate (∼1.0 m^3^/min) is 100 times higher than that of common organic membrane based filters (Li, 2011). While only up to 10% of the sampled biomass could be extracted by commercial kits, due to the swelling character of glass fibers. Such an actual sampling volume (150 m^3^ in 20 h) is acceptable for the detection of 16S rRNA genes, which present universally in all bacterial cells with multi copies(Lee et al., 2009).

However, the sampling volume has to be increased when the target gene of quantification is antibiotic resistant genes with concentrations significantly below the level of 16S rRNA genes. Actually, very few studies have been reported ARG contamination in bioaerosols.

Benefited from our novel methods, the actual sampling efficiency was significantly improved, indicated by the enhanced DNA yield shown in **Table 3** (Ling et al., 2013; Luhung et al., 2015; McEachran et al., 2015). Approximately 300 and 5000 ng DNA was recovered from clean and polluted air environment, respectively, which was sufficient for the qRT-PCR based 16S rRNA and functional gene quantification.

**Table 3:** Improved detection sensitivity of microbial contaminants by the application of MNP assisted biomass separation.

Locations	Volume based DNA yield (ng per m^3^)	Total DNA yield (ng per sample)	Gene concentration (copies per m^3^)		Reference
			16S rRNA gene	Functional gene (e.g., ARGs)	
Polluted air					
Live poultry trade market	∼20.0	4.93E+03	2.47E+07	3.13E+07 (*tetC*)	This study
Concentrated animal feeding operations	∼0.16^∗^	2.78E-01	8.20E+04	5.14E+03 (*tetW*)	Ling et al., 2013
Cattle feed yards	∼1.5^∗^	4.46E+01	1.90E+06	3.80E+06 (*tetM*)	McEachran et al., 2015
Clean air					
Urban air (streets, Shenzhen, China)	∼0.2	2.94E+02	2.57E+05	7.39E+02 (*tetC*)	This study
Urban air (campus, Singapore)	∼0.15	3.46E+00	1.90E+05^∗^	–	Luhung et al., 2015
Clinic indoor air	∼0.0001^∗^	4.96E-04	1.17E+02	1.02E+01 (*tetX*) 1.58E+01 (*tetW*)	Ling et al., 2013

*^∗^Estimated from the published data.*

In Ling’s study, the extracted DNA should be concentrated either by evaporation or ethanol precipitation before qPCR quantification based on their low absolute concentration [∼10 copies/m^3^ (*tetX*)] and small sampling volume (∼5.0 m^3^) (Ling et al., 2013). These concentrating procedures may induce additional errors to the overall measurement because losses are possible from inefficient precipitation and collection. Moreover, an increased PCR inhibition caused by concentrating contaminants in the extract is another important concern. Even in cases of a heavily polluted environment (Just et al., 2012) where the MDL is already satisfied, there are still technical demands for the improvement of the actual sampling rate by our novel method. A 10-folds increase in sampling rate indicates the sampling duration could be significantly shortened. Thus, a more precise time-course dynamic change of microbial compositions could be revealed (Qian et al., 2012).

Apart from the current application in biomass separation from glass fibers, the MNP based methods raised in the current study may have wider applications [e.g., the detection of sand adsorbed pathogens in beach health monitoring (Gu et al., 2003)]. Additionally, there are increasing studies using bimolecular functionalized NPs (e.g., Aptamers (Chen et al., 2008; Vikesland and Wigginton, 2010) or vancomycin (Gu et al., 2003; Neethirajan et al., 2014) functionalized NPs) for the capturing or sensing of pathogenic bacteria. During these detections, the non-specific bacterial adsorption on NPs was always underestimated or even neglected(Vikesland and Wigginton, 2010). According to our results, the buffering condition and surface coating are effective means for the tuning of non-specific adsorption, and have significant implications for the optimization of bacterial affinity materials.

## Conclusion

An MNP assisted biomass separation methods was developed that significantly improved the detection of airborne microbial contaminants sampled by TSP samplers. Benefited from our novel method, the overall DNA yield was increased by 10-folds, and a consistent recovery for the majority of airborne bacteria was demonstrated. This technique is convenient and promising in airborne microbial contaminants detection and feasible for application in environmental monitoring and other related fields. Furthermore, by in-depth comparison of the separated microbial community structure and surface charge was shown as the deciding factor for the interactions between MNPs and microorganisms, which is instructive for the development of materials with high microbial selectivity.

## Author Contributions

All authors listed, have made substantial, direct and intellectual contribution to the work and approved it for publication. X-LG and M-FS drafted the manuscript and contributed equally to this work.

## Conflict of Interest Statement

The authors declare that the research was conducted in the absence of any commercial or financial relationships that could be construed as a potential conflict of interest.
