# Supplementary material for: Non-selective Separation of Bacterial Cells with Magnetic Nanoparticles Facilitated by Varying Surface Charge
Source: Front Microbiol. 2016 Dec 1;7:1891. doi: 10.3389/fmicb.2016.01891 (PMC5130997; doi:10.3389/fmicb.2016.01891)
Supplement: Supplementary file 1 [file Data_Sheet_1.DOCX]

**Non-selective Separation of Bacterial Cells with Magnetic Nanoparticles Facilitated by Varying Surface Charge**

Xin-Lei Gao^1^, Ming-Fei Shao^1^*, Yi-Sheng Xu^2^*, Yi Luo^3^, Kai Zhang^1^, Feng Ouyang^1^, Ji Li^1^*

1. Shenzhen Graduate School, State Key Laboratory of Urban Water Resource and Environment, Shenzhen Key Laboratory of Water Resource Utilization and Environmental Pollution Control, Harbin Institute of Technology, Shenzhen 518055, China
2. State-Key Laboratory of Chemical Engineering, East China University of Science and Technology, Shanghai, 200237, China
3. College of Environmental Science and Engineering, Ministry of Education Key laboratory of Pollution Processes and EnvironmentalCriteria, Nankai University, Tianjin 300071, China

*Corresponding author

Ming-Fei Shao：

Tel./fax: +86-755-6032692, e-mail: mfshao@hitsz.edu.cn；

Yi-Sheng Xu:

e-mail: yshxu@ecust.edu.cn

Ji Li：

Tel./fax: +86-755-26032692, e-mail: Liji99@hitsz.edu.cn；

**Caption of Tables and Figures in Supporting Information**

Table S1. Sampling imformations.

Table S2. qPCR Primers.

Figure S1. The TEM image of (a) Fe_3_O_4_ MNPs and (b) Fe_3_O_4_ @SiO_2_ core-shell MNPs.

Figure S2.The FTIR spectra of (a) Fe_3_O_4_ @SiO_2_ core-shell MNPs and (b)Fe_3_O_4_ MNPs.

Figure S3. The rarefaction curve of airborne microbial community collected in wet market environment.

Figure S4. PCoA analysis showing the relatedness of treatment and control samples based on the compositions of whole microbial community.

Table S1. Sampling imformations

| Sample  Name | Sampling Date | Description | Volume  (m^3^) | Sampling duration  (min) |
| --- | --- | --- | --- | --- |
| LPT 1 | 5/28/2015 | Live poultry trade area  of the market | 252 | 240 |
| LPT 2 | 5/28/2015 |  |  |  |
| LPT 3 | 6/02/2015 |  |  |  |
| US 1 | 5/29/2015 | Urban  street side | 1449 | 1380 |
| US 2 | 5/30/2015 |  |  |  |
| US 3 | 6/06/2015 |  |  |  |

Table S2. qPCR Primers

| **Gene** | **Primer sequences** | **Amplicon Size** | **Annealing temperature (°C)** | **Reference** |
| --- | --- | --- | --- | --- |
| *tetC* | CTTGAGAGCCTTCAACCCAG  ATGGTCGTCATCTACCTG | 418 | 55 | ([Zhang and Zhang 2011](#_ENREF_2)) |
| *16S rRNA* | CCTACGGGAGGCAGCAG(338F)  ATTACCGCGGCTGCTGG(518R) | 200 | 56 | ([Ling et al. 2013](#_ENREF_1)) |


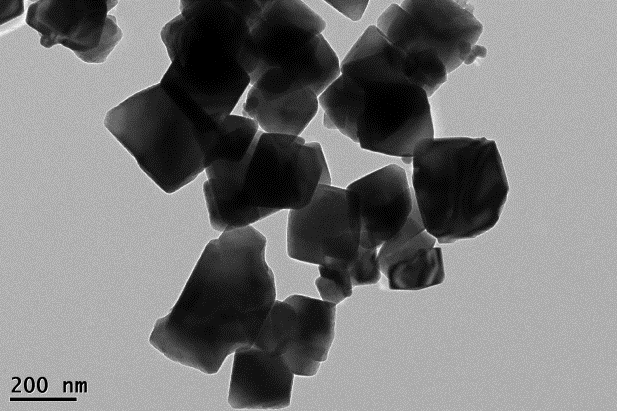

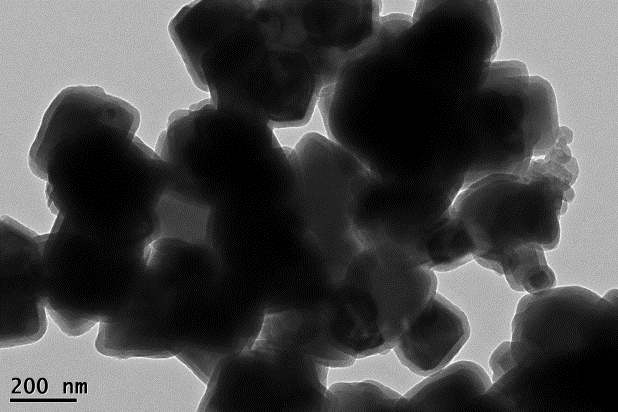


(a) (b)

Figure S1. The TEM image of (a) Fe_3_O_4_ MNPs and (b) Fe_3_O_4_ @SiO_2_ core-shell MNPs. The particle size of both types of MNPs were estimated to be 100~200 nm in diameter. A silica shell with its thickness of 10-20 nm was introduced after silica encapsulation.

| 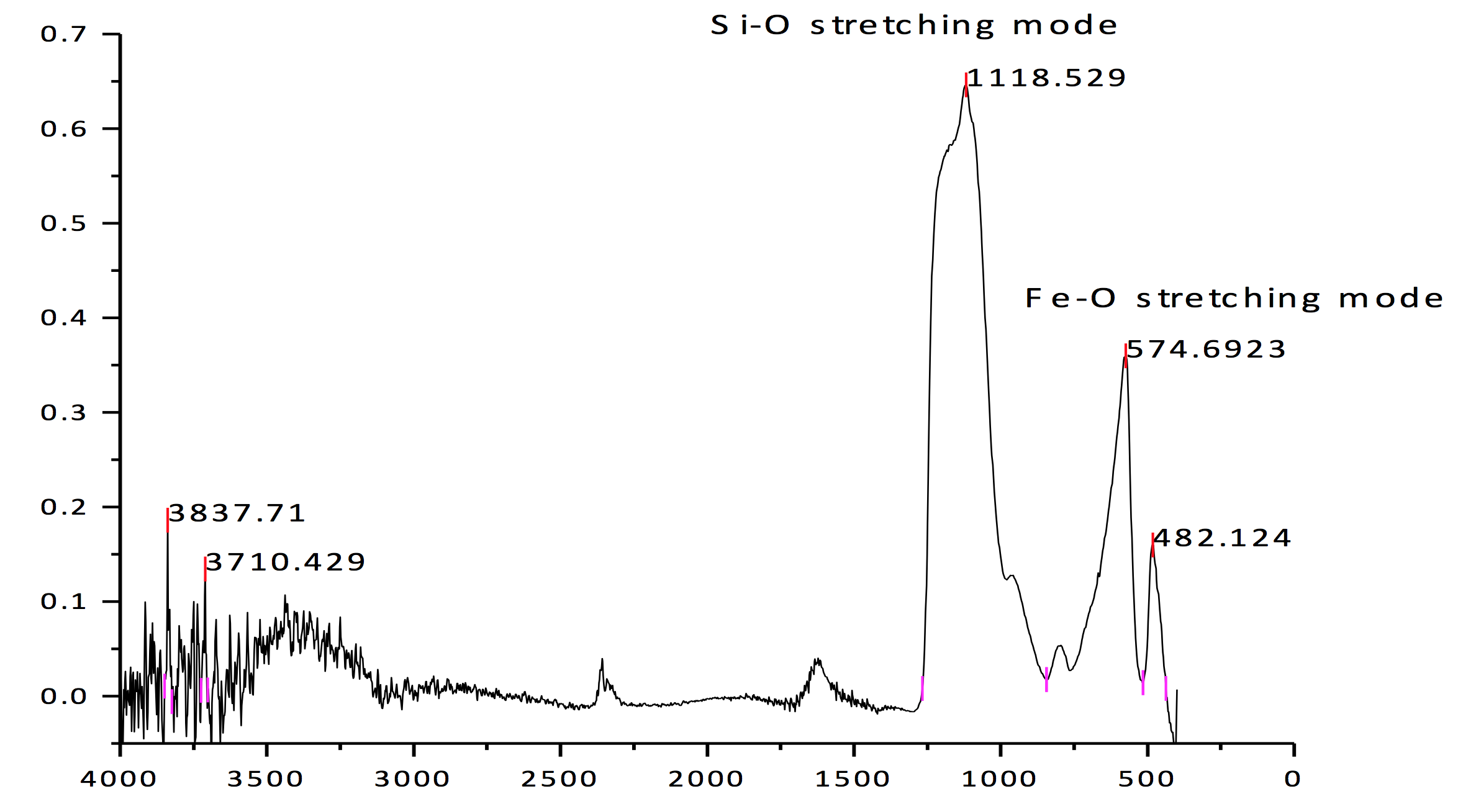(a) |
| --- |
| 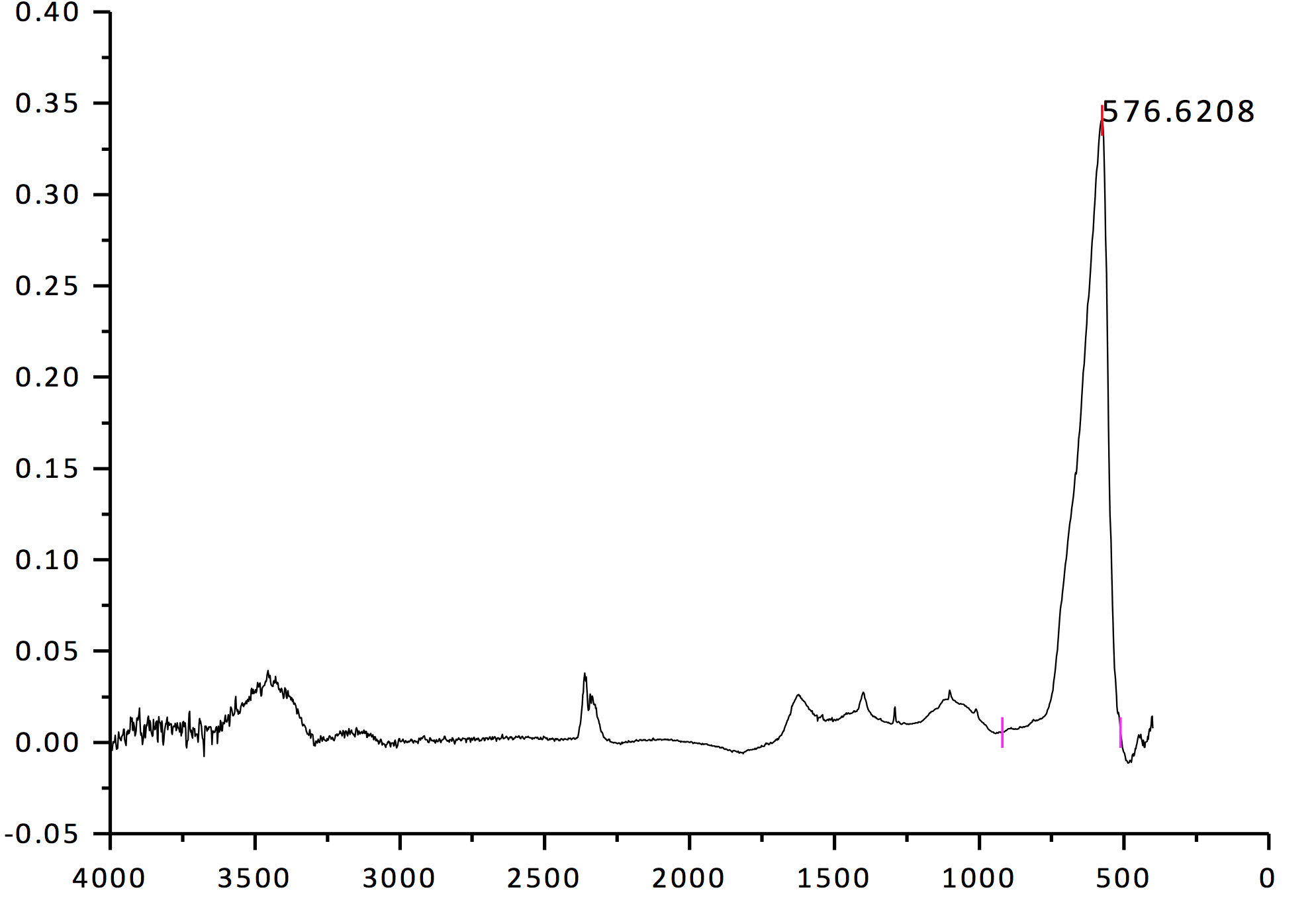(b) |

Figure S2.The FTIR spectra of (a) Fe_3_O_4_ @SiO_2_ core-shell MNPs and (b)Fe_3_O_4_ MNPs. Significant Si-O strenching at 1118 nm was revealed, indicating successful silica encapsulation.

Figure S3. The rarefaction curve of airborne microbial community collected in wet market environment.

Figure S4. PCoA analysis showing the relatedness of treatment and control samples based on the compositions of whole microbial community.

**REFERENCES**

Ling, A.L., Pace, N.R., Hernandez, M.T. and LaPara, T.M. (2013) Tetracycline Resistance and Class 1 Integron Genes Associated with Indoor and Outdoor Aerosols. Environmental Science & Technology 47(9), 4046-4052.

Zhang, X.X. and Zhang, T. (2011) Occurrence, Abundance, and Diversity of Tetracycline Resistance Genes in 15 Sewage Treatment Plants across China and Other Global Locations. Environmental Science & Technology 45(7), 2598-2604.
